# Surfactant-Assisted Synthesis of Micro/Nano-Structured LiFePO_4_ Electrode Materials with Improved Electrochemical Performance

**DOI:** 10.3390/ma15248953

**Published:** 2022-12-14

**Authors:** Yuqing Qiao, Ying Liu, Jianguo Zhu, Peng Jia, Liqiao Zhang, Wei Zhou, Tifeng Jiao

**Affiliations:** 1 Hebei Key Laboratory of Applied Chemistry, School of Environmental and Chemical Engineering, Yanshan University, Qinhuangdao 066004, China; 2 State Key Laboratory of Metastable Materials Science and Technology, Yanshan University, Qinhuangdao 066004, China

**Keywords:** li-ion batteries, LiFePO_4_, oleic acid, cathode material

## Abstract

As an electrode material, LiFePO_4_ has been extensively studied in the field of energy conversion and storage due to its inexpensive cost and excellent safety, as well as good cycling stability. However, it remains a challenge to obtain LiFePO_4_ electrode materials with acceptable discharge capacity at low temperature. Here, micro/nano-structured LiFePO_4_ electrode materials with grape-like morphology were fabricated via a facile solvothermal approach using ethanol and OA as the co-solvent, the surfactant as well as the carbon source. The structure and electrochemical properties of the LiFePO_4_ material were investigated with x-ray diffraction (XRD), field emission scanning electron microscopy (SEM), cyclic voltammetry (CV) and electrochemical impedance spectroscopy (EIS), and the formation mechanism of the self-assembled micro/nano-structured LiFePO_4_ was discussed as well. The micro/nano-structured LiFePO_4_ electrode materials exhibited a high discharge capacity (142 mAh·g^−1^) at a low temperature of 0 °C, and retained 102 mAh·g^−1^ when the temperature was decreased to −20 °C. This investigation can provide a reference for the design of micro/nano-structured electrode materials with improvement of the electrochemical performance at low temperature.

## 1. Introduction

Due to its high theoretical capacity (170 mAh·g^−1^), moderate voltage plateau (3.4 V versus Li^+^/Li), long cycling life, environmental compatibility and low cost [1,2,3,4], LiFePO_4_ (LFP) has been intensively investigated as one of the most promising cathode materials for rechargeable lithium-ion batteries used in electric vehicles (EVs) and hybrid electric vehicles (HEVs). However, LiFePO_4_ cathode material presents the lower li-ion diffusion coefficient (10^−14^~10^−16^ m^2^·S^−1^) [5,6,7] and electronic conductivity (10^−9^~10^−10^ S·cm^−1^) when compared with other cathode materials such as LiCoO_2_ [8], Li_3_V_2_(PO4)_3_/C [9], Cu_0.95_V_2_O_5_ [10], FeF_3_ [11], V_2_O_5_ [12]. It is reported that an effective approach to enhance electronic conductivity is to coat conductive materials on the surface of LiFePO_4_ particles or dope metal-ions/metal-oxide to change LiFePO_4_ lattice [13,14,15,16]. As the li-ion diffusion path (such as diffusion distance and diffusion channel) is predominantly controlled by the diffusion time (diffusion coefficient), nanocrystallization is favorable for improvement of the li-ion diffusion time for the short diffusion distance [17,18,19]. Peng et al. [20] synthesized a one-dimensional (1D) single-crystalline nanoarchitectures LiFePO_4_ with good rate capability. Zhao et al. [21] synthesized a two-dimensional (2D) single crystalline LiFePO_4_ with the highest pore density for lithium-ion insertion/extraction. Xia et al. [22] prepared large-scale LiFePO_4_ microspheres with a three-dimensional (3D) porous microstructure and these micro/nano-structured LiFePO_4_ microspheres have a high tap density, which show excellent rate capability and cycle stability as electrodes.

In addition, as one of the most widely used electrode materials of lithium-ion batteries (LIBs) for electric vehicles, LiFePO_4_ cathode materials possess many excellent properties, including excellent safety [1,2,3,4,22,23,24,25,26,27,28]. However, the poor discharge capacity at relative lower temperatures (below −20 °C) hinders its practical applications in special environment or regions. Generally, the working temperature of LiFePO_4_ cathode materials is between −10 and 55 °C. For LiFePO_4_ cathode materials used at low temperatures, several problems should be considered. First, the specific capacity of LiFePO_4_ cathode materials will reduce sharply (40~60% at a low temperature of −20 °C); Second, the capacity of LiFePO_4_ cathode materials will be consumed rapidly at a low temperature. In addition, lithium dendrites will be formed easily when the LIBs are charged at low temperatures, which can pierce the separator between electrodes and cause an inner short circuit [27]. Cao et al. [27] reported that kinetic characteristics of LIBs determine their electrochemical performance at low temperatures. To improve the dynamic properties, a number of LiFePO_4_ electrode materials with nano scales or with a porous structure have been developed [2,20,22,26].

To date, a number of synthesized methods, such as solid-state techniques [23], hydrothermal synthesis [24], co-precipitation [25], sol-gel reaction [26], as well as other methods with improved electrochemical properties [27,28], have been developed to prepare nano-sized LiFePO_4_ particles. As a typical surfactant, OA is used as surfactant and template in solvothermal synthesis of LiFePO_4_ cathode materials. Yang et al. [29] reported that the introduction of OA can lead to a smaller particle size and a homogeneous size distribution of LiMnPO_4_ particles, resulting in improved electrochemical performance. In addition, Rangappa et al. [30] prepared LiFePO_4_ cathode materials with a flower-like microstructure using a solvothermal method with ethylene glycol (EG) and OA as co-solvent and surfactant, where the EG and OA played a key role in controlling the size and morphology of LiFePO_4_ nanocrystals. In addition, the EG and OA also acted as the carbon source when the precursor was carbonized at a high temperature (600 °C). The nano-scaled LiFePO_4_ cathode materials exhibited a high specific capacity and a good cyclic performance. In this work, micro/nano-structured LiFePO_4_ electrode materials with grape-like morphology were fabricated via a facile solvothermal approach using ethanol and OA as the co-solvent, the surfactant as well as the carbon source. The micro/nano-structured LiFePO_4_ electrode materials exhibited a high discharge capacity of 142 mAh·g^−1^ at 0 °C, and retained 102 mAh·g^−1^ when the temperature was decreased to −20 °C. This investigation can provide a reference for the design of micro/nano-structured LiFePO_4_ electrode materials with improvement of the electrochemical performance at a low temperature.

## 2. Experiment

### 2.1. Materials

LiFePO_4_ microspheres were synthesized using a facile co-solvothermal method with ethanol, deionized water as the co-solvent and OA as surfactant. All the reagents used for synthesis and experiments were analytical grade and employed as received, without any further purification. First, 40 mL ethanol were added into a beaker and mixed with 20 mL deionized water. Designed amount of OA (C_18_H_34_O_2_, approx. 2.0 mL, Tianjin Kaitong chemical Co., Ltd., Tianjin, China) was added in the mixed solvent and heated to be dissolved. The mixture was separated into two parts evenly (beaker A and beaker B), and then 12 mmol Lithium hydroxide (LiOH·H_2_O 90%, Tianjin Fengchuan chemical Co., Ltd., Tianjin, China) was added into beaker A, followed by adding 3 mmol phosphoric acid (H_3_PO_4_ 85%, Synth) to form a white-color suspension. At the same time, 3 mmol Iron sulfate (FeSO_4_·H_2_O 99.9%, Xilong chemical Co., Ltd., Shantou, China) was added into beaker B, and then 0.5 mmol of ascorbic acid (99.7%, Xilong chemical Co., Ltd.) was added into beaker B synchronization to protect Fe^2+^ from being oxidized. Next, the mixed solution in beaker B was dropped into beaker A under vigorous magnetic stirring for 30 min, forming an absinthe-green suspension with large amounts of precipitates, and a pH value (approx. 7) for the absinthe-green mixture was controlled by adding hydrazine hydrate solution. Subsequently, the mixture was transferred into a 100 mL Teflon-stainless autoclave and heated at 180 °C for 18 h. Following, the mixture, after cooling down to room temperature, was centrifuged to obtain the precipitation, and then the precipitation was washed and centrifuged with ethyl alcohol and deionized water for several times to get rid of the remaining ions. The precursors were treated at 700 °C for 6 h in a N_2_ atmosphere in a tube furnace with a heating-rate of 3 °C/min to obtain the LiFePO_4_ electrode materials with OA. To investigate comparatively, the LiFePO_4_ electrode material was also prepared at the same condition without OA.

### 2.2. Physiochemical Characterization of LiFePO_4_

Crystal structures of the micro/nano-structured LiFePO_4_ electrode materials with grape-like morphology were determined using a Rigaku D/max 2500 pc X-ray diffractometer (XRD), utilizing a Cu Kα radiation source (λ = 0.15406 nm) operated at 40 kV and 30 mA with a step scan of 0.02° (10–80°, 4° min^−1^). The microstructure and the morphology of the prepared LiFePO_4_ mounted on a Cu grid were characterized with a JEM-2100 transmission electron microscope (TEM) and S-4800 field-emission scanning electron microscopy (SEM). Raman spectrum was performed on a Renishaw Gloucestershire with a laser wavelength of 514 nm.

### 2.3. Electrochemical Measurement

The electrochemical performances of the micro/nano-structured LiFePO_4_ electrode materials with grape-like morphology were measured using two-electrode cells assembled in an Ar-filled glove box. The working electrodes were prepared by mixing the activated material (as-prepared LiFePO_4_ electrode materials) with acetylene black and polovinylidene fluoride (PVDF) at a mass ratio of 80:15:5 by blending in N-methylpyrrolidone to form a slurry; then, the slurry was spread onto an aluminum foil homogeneously to obtain the working electrode. The as-prepared working electrode was then dried at 120 °C for 12 h in a vacuum drying oven. Lithium foil was used as the anode in a mixture electrolyte solution (1 M LiPF_6_: ethylene carbonate (EC): diethyl carbonate (DEC) = 1:1:1).

The electrochemical performances of the micro/nano-structured LiFePO_4_ electrode materials were carried out with a BTS 5 V, 1 A system under different current densities, with voltage ranging from 2.5 to 4.3 V. A cyclic voltammetry (CV) test (scan rate was 0.4 mV·S^−1^) was performed. Electrochemical impedance spectroscopy (EIS) was used for measuring the charge transfer resistance (R_ct_) and investigate the beneficial effect of micro/nano-structured LiFePO_4_ on battery reaction kinetics. After the test electrodes were completely activated, EIS measurements were conducted on a CHI660E electrochemical workstation with ZPLOT electrochemical impedance software. The EIS spectra of the micro/nano-structured LiFePO_4_ electrode materials were obtained in a frequency range from 100 kHz to 0.01 Hz with an alternating current amplitude of 5 mV (a sinusoidal excitation voltage of 5 mV). According to the analysis model [22,31], an equivalent circuit for the LiFePO_4_ electrode materials was used. Using least-square method, the parameters in the equivalent circuit were fitted with ZVIEW electrochemical impedance software. All potentials cited in this paper are referred to Li/Li^+^.

## 3. Results and Discussion

### 3.1. Characterization of Structure and Morphology

**Figure 1:**
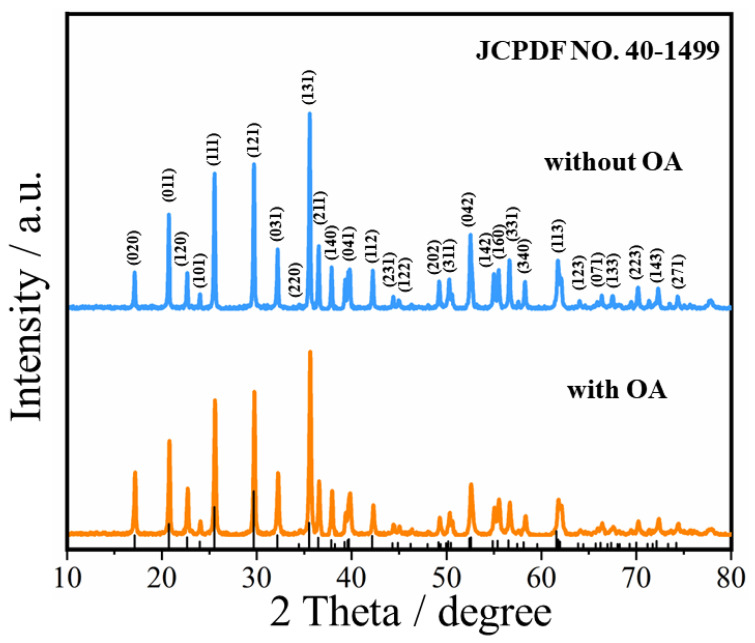
XRD patterns of LiFePO_4_ prepared without and with OA.

Figure 1 shows the XRD patterns of the as-prepared LiFePO_4_ cathode materials without and with OA used as the surfactant, respectively. Positions of the Bragg peaks on the XRD pattern of the as-prepared LiFePO_4_ cathode materials with OA match well with the XRD pattern of the standard diffraction lines of the identified phase, indicating a good phase matching with the LiFePO_4_ (JCPDS # 40-1499) with an orthorhombic olivine-type structure. In addition, the lattice parameter and cell volume of the LiFePO_4_ cathode materials are calculated with JADE 5.0 software, respectively, and the results are given in Table 1. We note that those lattice parameters of the LiFePO_4_ cathode materials prepared with OA are larger than that LiFePO_4_ cathode materials prepared without OA.

**Figure 2:**
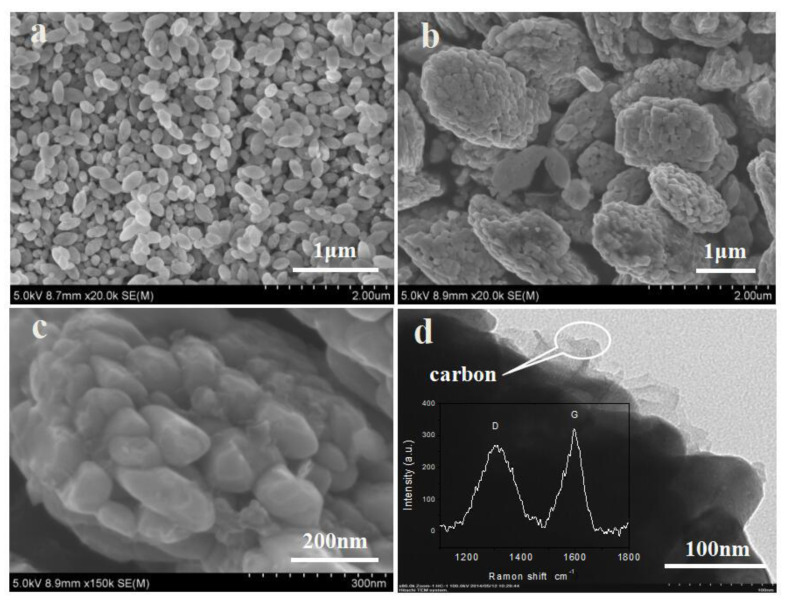
SEM images of LiFePO_4_ prepared without OA (**a**) and with OA (**b**), high magnification (**c**), TEM with an inset of Raman result LiFePO_4_ microspheres prepared with OA (**d**).

Figure 2 is SEM images and TEM images of the LiFePO_4_ particles prepared. As seen from Figure 2a, the LiFePO_4_ particles prepared without OA are dispersed with homogeneous single-particles, while the LiFePO_4_ particles prepared with OA consist of spherical particles with a grape-like morphology (Figure 2b). At higher magnification (see Figure 2c), the grape-like LiFePO_4_ microspheres are self-assembled by a number of nano particles with an average diameter of about 100 nm. Two peaks were observed on the Raman spectra (inset in Figure 2d). The former is the signal of defective carbon atoms (sp^3^-boned, 1340–1350 cm^−1^) and the latter is the signal of carbon atoms with sp^2^-bonded carbon atoms (graphite carbon, 1580–1600 cm^−1^). It is seen from the inset in Figure 2d that a number of thin film carbon inlaid on the surface of the LiFePO_4_ particles prepared, which coincides well with Raman’s analysis.

### 3.2. Formation Mechanism

**Figure 3:**
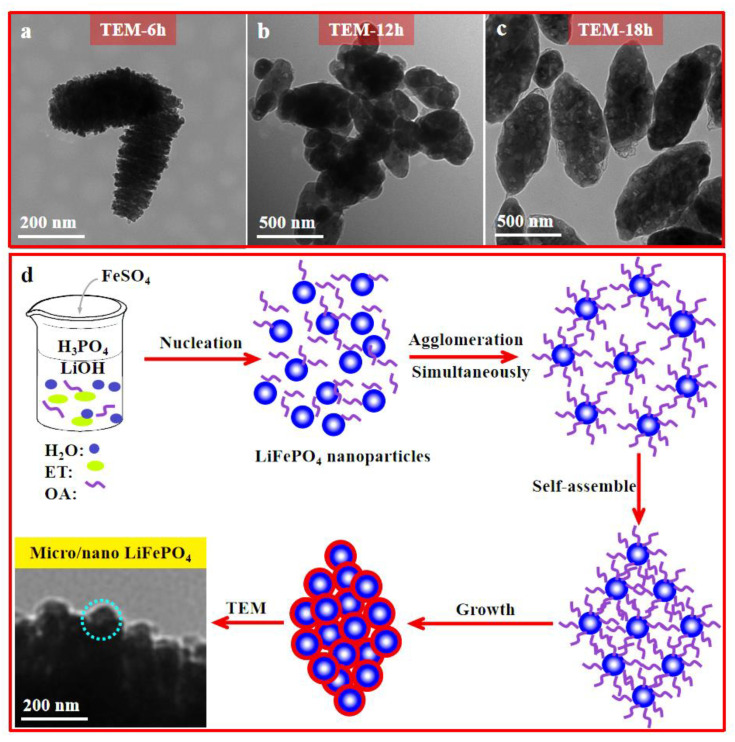
TEM images of LiFePO_4_ microspheres with OA at 6 h (**a**), 12 h (**b**), 18 h (**c**) and the schematic illustration of formation mechanism of the grape-like LiFePO_4_ microsphere (**d**).

To explore the formation mechanism of a grape-like LiFePO_4_ microsphere, the effect of heating time on the morphology of the micro/nano-structured LiFePO_4_ electrode material is discussed in more details. Figure 3a–c shows the TEM images of the micro/nano-structured LiFePO_4_ material synthesized after a certain times. As shown in Figure 3a, the LiFePO_4_ electrode materials with irregular morphology after 6 h were assembled with a large numbers of LiFePO_4_ nanocrystals. When the heating time extended to 12 h, the quasi grape-like LiFePO_4_ microspheres were observed (Figure 3b), and when the heating time extended to 18 h, grape-like LiFePO_4_ microspheres were observed clearly (Figure 3c).

Xia et al. [22] prepared spindle-like LiFePO_4_ nanocrystals and investigated the growth mechanism of the LiFePO_4_, indicating that reaction time and the pH environment played multifold roles in controlling the spindle-like morphology of LiFePO_4_. In addition, the growth orientation and the grain boundaries were also investigated, indicating that the spindle-like LiFePO_4_ nanocrystals were assembled with the growth process of LiFePO_4_ nanocrystals happening simultaneously. Rangappa et al. [30] prepared flower-like LiFePO_4_ nanocrystals using a solvothermal method with ethylene glycol (EG) and OA as co-solvent and surfactant, where the EG and OA acted as a soft template in directing the growth of LiFePO_4_ nanocrystals at the early stages and the flower-like structure was self-assembled in the presence of OA. According to the growth mechanism proposed by Rangappa et al., it was the interaction of the adsorbed OA molecules that played an important role in controlling the size and flower-like morphology of LiFePO_4_ nanocrystals.

The formation mechanism of the LiFePO_4_ microspheres is schematically illustrated in Figure 3d [22,30]. First, LiFePO_4_ precursor was synthesized with the mixing of the reactants in the co-solvent system; OA, as a typical surfactant, is composed of a hydrophilic head (carbonyl group (C=O)) and a hydrophobic organic tail (−(CH_2_)_7_CH=CH (CH_2_)_7_CH_3_). Due to the hydrophilic of the LiFePO_4_ precursor, the hydrophilic head of OA tends to adsorb on the surface of LiFePO_4_ with the hydrophobic organic tail outward and those hydrophobic organic tails are aggregated after heat treatment. In this work, micro/nano-structured LiFePO_4_ electrode materials with grape-like morphology were fabricated via a facile solvothermal approach using ethanol and OA as the co-solvent, the surfactant as well as the carbon source, where the OA played a key role in controlling morphology of LiFePO_4_ nanocrystals.

### 3.3. Electrochemical Properties

**Figure 4:**
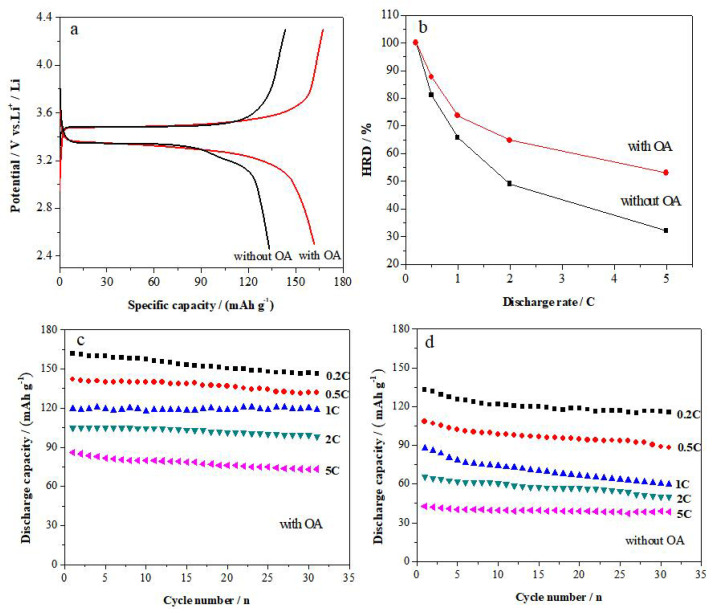
Initial charge/discharge profiles (**a**), the HRD (**b**), cycle performance of LiFePO_4_ electrodes prepared with OA (**c**) and without OA (**d**).

Figure 4a shows the initial charge/discharge profiles for the LiFePO_4_ electrode prepared without and with OA. As seen from Figure 4a, the LiFePO_4_ prepared with OA has a specific discharge capacity of 161 mAh·g^−1^ at 0.2 C, which is increased by 20.9% in comparison with that of the LiFePO_4_ synthesized without OA (133 mAh·g^−1^).

The relationship between the discharge capacity and the discharge current density of the LiFePO_4_ cathode materials at 0.2 C, 0.5 C, 1 C, 2 C and 5 C is shown in Figure 4b. It is learnt from Figure 4b that the high-rate discharge capacity (HRD) of the LiFePO_4_ cathode materials prepared with OA is 142, 119, 105 and 86 at 0.5 C, 1 C, 2 C and 5 C, which is about 31%, 36%, 61% and 101% higher than that of LiFePO_4_ materials prepared without OA, respectively. Figure 4c,d depicts the cycling stability at 0.2 C, 0.5 C, 1 C, 2 C and 5 C of the LiFePO_4_ cathode materials. It is obvious that all the capacity retention at 30 times charge/discharge cycles exceeds 90%.

**Figure 5:**
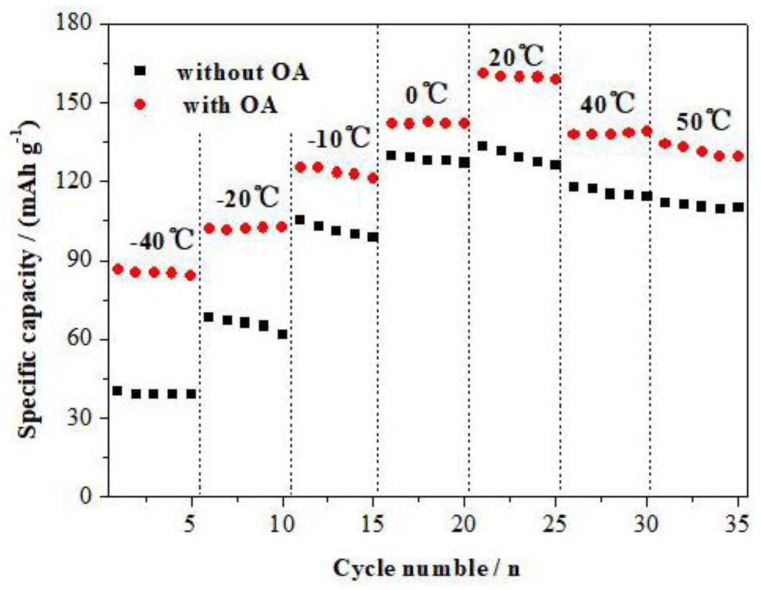
Effect of temperature on the specific capacity of LiFePO_4_ electrodes prepared.

Figure 5 shows the specific discharge capacity of the LiFePO_4_ electrode prepared at the temperatures ranging from −40 °C to 50 °C. Evidently, the LiFePO_4_ cathode materials prepared with OA has higher discharge capacity than that without OA at all the temperatures. At 0 °C, −10 °C, −20 °C and −40 °C, the specific discharge capacity is 142 mAh·g^−1^, 125 mAh·g^−1^, 102 mAh·g^−1^ and 87 mAh·g^−1^, which is respectively 88%, 78%, 63% and 54% when compared with the specific discharge capacity of 161 mAh·g^−1^ at the ambient temperature (20 °C).

**Figure 6:**
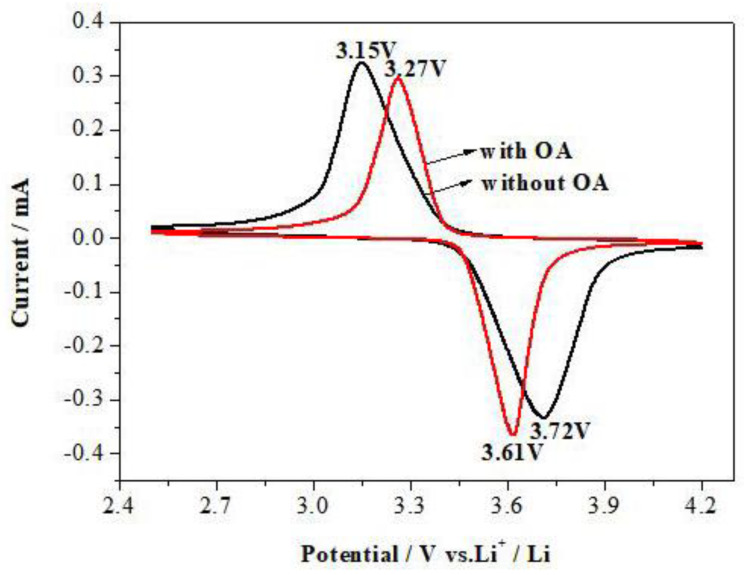
Cyclic voltametric profiles of LiFePO_4_ electrodes prepared.

Figure 6 shows the cyclic voltammetry (CV) curves of the samples at the scan rate of 0.4 mV·S^−1^ after activation for 2 cycles. It can be seen from Figure 6 that the oxidation peaks and reduction peaks of the sample prepared with OA appear at 3.27 V and 3.61 V with a gap of 0.34 V, which is less than that of the sample prepared without OA (0.57 V).

**Figure 7:**
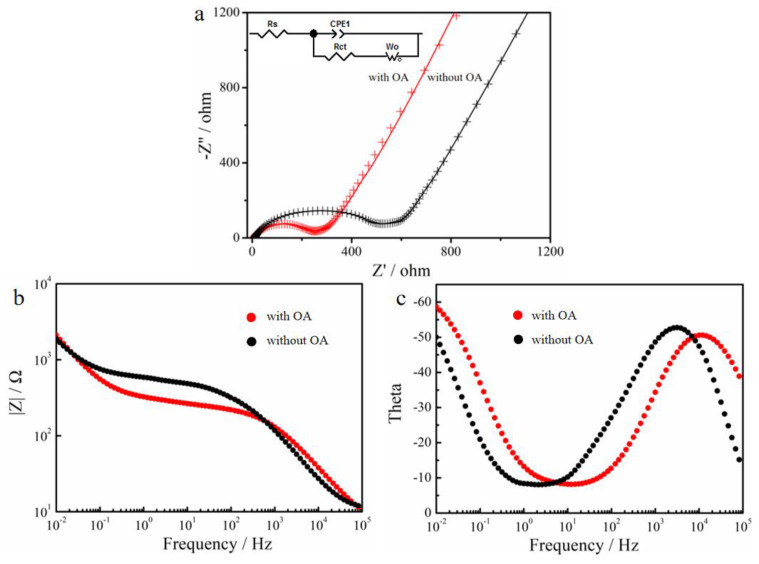
Impedance spectra profiles of LiFePO_4_/C electrodes prepared. (**a**) Nyquist diagrams (The inset is an equivalent circuit diagram). (**b**,**c**) Bode plots.

To further examine the electrode’s behavior of the micro/nano-structured LiFePO_4_, EIS measurements were performed and the EIS spectra of the micro/nano-structured LiFePO_4_ electrode materials were obtained in a frequency range from 100 kHz to 0.01 Hz (Figure 7). An equivalent circuit for the micro/nano-structured LiFePO_4_ electrode materials was used to analyze the impedance spectra (inset in Figure 7a), where the R_s_, R_ct_, CPE and W_o_ represent the resistance of the electrolyte (R_s_), the charge–transfer resistance (R_ct_), the double-layer capacitance and the Warburg impedance, respectively. In high frequency, an intercept at the *Z*’ axis was used to evaluate the R_s_. In the high-middle frequency, the semicircle was used to evaluate the R_ct_. In the low frequency, the inclined line was used to evaluate the W_o_, which was attributed to the diffusion of Li ion into the micro/nano-structured LiFePO_4_ electrode materials. The equivalent capacitance is used to describe the electric double layer between the electrode and the solution. However, the impedance behavior of the solid electrode’s electric double layer deviates somewhat from that of the equivalent capacitance. This phenomenon is generally called “dispersion effect”. The dispersion effect caused by the material surface inhomogeneity of micro/nano structures is called constant phase angle element (CPE). The CPE is related to changes in the thickness, roughness and porosity of the electrode material. The fitting result indicates that the value of R_s_ is 5.3 Ω for the micro/nano-structured LiFePO_4_ electrode, which is about 43% lower than that of LiFePO_4_ electrode prepared without OA, indicating a smaller resistance of the electrolyte. The fitting result indicates that the value of R_ct_ is 221 Ω for the micro/nano-structured LiFePO_4_ electrode, which is about 50% lower in comparison with that of the LiFePO_4_ electrode materials prepared without OA (444 Ω) (Figure 7a, Table 2). Meanwhile, we add bode plots to support the discussion on Nyquist diagrams (Figure 7b,c). In addition, the straight line in low frequency was attributed to the diffusion of li ion into the bulk of the LiFePO_4_ electrode materials, where the slope of the straight line represented the Warburg impedance. It is observed that the Warburg impedance of the micro/nano-structured LiFePO_4_ electrode materials is higher in comparison with the other LiFePO_4_ electrode materials. The parameters of the equivalent circuit for the micro/nano-structured LiFePO_4_ electrode materials and the LiFePO_4_ electrode materials prepared without OA were recorded in Table 1.

## 4. Conclusions

In this investigation, micro/nano-structured LiFePO_4_ electrode materials with grape-like morphology were fabricated via a facile solvothermal approach using mixture of ethanol/water/oleic acid as a co-solvent and oleic acid (OA) as a surfactant. We note that those lattice parameters of the LiFePO_4_ cathode materials prepared with OA are larger than that LiFePO_4_ cathode materials prepared without OA. The micro/nano-structured LiFePO_4_ electrode materials exhibited a high discharge capacity of 142 mAh·g^−1^ at 0 °C, and retained 102 mAh·g^−1^ when the temperature was decreased to −20 °C. The micro/nano-structured LiFePO_4_ electrode materials revealed a lower charge–transfer resistance, indicating an improved dynamic performance of li-ion diffusion.

## Figures and Tables

**Table 1:** Some XRD and electrochemical parameters of the LiFePO_4_ electrodes prepared without and with OA.

Samples	*a* (nm)	*b* (nm)	*c* (nm)	V (nm^3^)	*C*_20_ (mAh·g^−1^)	*C*_−20_ (mAh·g^−1^)	R_ct_ (Ω)
without OA	1.0322	0.6002	0.4696	0.2909	133	68	444
with OA	1.0338	0.6007	0.4702	0.2916	161	102	221

*C*_20_ (mAh·g^−1^): the capacity at 20 °C; *C_−_*_20_ (mAh·g^−1^): the capacity at −20 °C; R_ct_ (Ω): the charge–transfer reaction resistance.

**Table 2:** The specific values of each parameter in the equivalent circuit.

	R_s_	CPE	R_ct_	Chi-Squared
with OA	5.3	8.7 × 10^6^	221	9.7 × 10^4^
without OA	9.3	1.3 × 10^5^	444	1.7 × 10^3^

## Data Availability

Data presented in this article are available on request from the corresponding author.
